# Regulation of hematopoietic and leukemia stem cells by regulatory T cells

**DOI:** 10.3389/fimmu.2022.1049301

**Published:** 2022-11-02

**Authors:** Carsten Riether

**Affiliations:** ^1^ Department of Medical Oncology, Inselspital, Bern University Hospital, University of Bern, Bern, Switzerland; ^2^ Department for BioMedical Research (DBMR), University of Bern, Bern, Switzerland

**Keywords:** regulatory T cell (Treg), leukemia stem cell (LSC), immune escape, hematopoietic stem cell, hematopoietic stem cell niche

## Abstract

Adult bone marrow (BM) hematopoietic stem cells (HSCs) are maintained in a quiescent state and sustain the continuous production of all types of blood cells. HSCs reside in a specialized microenvironment the so-called HSC niche, which equally promotes HSC self-renewal and differentiation to ensure the integrity of the HSC pool throughout life and to replenish hematopoietic cells after acute injury, infection or anemia. The processes of HSC self-renewal and differentiation are tightly controlled and are in great part regulated through cellular interactions with classical (e.g. mesenchymal stromal cells) and non-classical niche cells (e.g. immune cells). In myeloid leukemia, some of these regulatory mechanisms that evolved to maintain HSCs, to protect them from exhaustion and immune destruction and to minimize the risk of malignant transformation are hijacked/disrupted by leukemia stem cells (LSCs), the malignant counterpart of HSCs, to promote disease progression as well as resistance to therapy and immune control. CD4^+^ regulatory T cells (Tregs) are substantially enriched in the BM compared to other secondary lymphoid organs and are crucially involved in the establishment of an immune privileged niche to maintain HSC quiescence and to protect HSC integrity. In leukemia, Tregs frequencies in the BM even increase. Studies in mice and humans identified the accumulation of Tregs as a major immune-regulatory mechanism. As cure of leukemia implies the elimination of LSCs, the understanding of these immune-regulatory processes may be of particular importance for the development of future treatments of leukemia as targeting major immune escape mechanisms which revolutionized the treatment of solid tumors such as the blockade of the inhibitory checkpoint receptor programmed cell death protein 1 (PD-1) seems less efficacious in the treatment of leukemia. This review will summarize recent findings on the mechanisms by which Tregs regulate stem cells and adaptive immune cells in the BM during homeostasis and in leukemia.

## Introduction

During the last decade a series of studies have been performed to decipher the role and contribution of non-hematopoietic (osteoblasts, stromal and endothelial cells, pericytes, Schwann cells and sympathetic neurons) as well as mature hematopoietic cells to the hematopoietic stem cell (HSC) niche in the bone marrow (BM). All these different cell types were allocated as components of the HSC niche and even as critical regulators of HSC function. Especially intensive work on cells of non-hematopoietic origin by many different groups unraveled the composition of the HSC niche and the cellular and molecular interactions exchanged between its different stromal and endothelial components and their contribution to HSC function (1). Recent work has highlighted the importance of mature immune cells as constituents of the HSC niche (1). Regulatory T cells (Tregs) have been initially identified as a specialized T cell subpopulation which suppresses immune responses to guarantee immune homeostasis and self-tolerance. Recently, however, Tregs have been shown to also control non-lymphoid processes, insulin resistance in visceral fat, muscle repair and to prevent lung damage (2). In the BM microenvironment Treg frequencies are higher than in other secondary lymphoid organs suggesting a role for Tregs in shaping an immunosuppressive BM microenvironment which may protect HSCs from elimination and damage. However, the current understanding of Tregs in the regulation of HSCs and/or the HSC niche under steady state conditions is still limited. During leukemia development, the immune microenvironment in the BM undergoes massive changes with especially immunosuppressive Tregs infiltrating or accumulating. These Tregs may be able to directly promote leukemogenesis or to protect LSCs from elimination by activated immune cells. However, like for HSCs, the actual interaction of Tregs with LSCs remains in part elusive. Therefore, in this review we discuss recent insights into the communication between Tregs, HSCs and LSCs during health and hematological disease.

## The perivascular HSC niche in the BM

Under homeostatic conditions, the majority of HSCs in the BM are in a quiescent state to prevent HSC exhaustion and to maintain and protect their self-renewal capacity (1). Fundamental HSC characteristics such as self-renewal, differentiation and proliferation are crucially regulated by hematopoietic and stromal components of the HSC niche. Perivascular regions in the BM form distinct niches and have been identified to regulate HSC quiescence and the supply of lineage-committed progenitors (1). Especially NG2-expressing pericytes maintain HSCs in a quiescent state as depletion of NG2-expressing cells resulted in cycling of HSCs, loss of long-term repopulating HSCs and altered HSC localization in the BM. In contrast, cycling HSCs are found near sinusoids and more specifically to sinusoids associated with stem cell factor (SCF) - producing leptin receptor-positive (LEPR^+^) cells. Depletion of SCF in LEPR^+^ cells reduced BM HSC numbers (3). Consequently, it is postulated that once entering cell cycle quiescent HSCs translocate from a NG2^+^ periarteriolar to a LEPR^+^ perisinusoidal region of the HSC niche. In addition, the low oxygen-permeability of arteriolar blood vessels in the BM promotes HSC quiescence by reducing the accumulation of reactive oxygen species (ROS) in HSCs in contrast to highly oxygen-permeable sinusoids which confer proliferation and differentiation signals to the HSCs (4–6). Furthermore, endothelial cells have been identified together with LEPR^+^ cells as the main sources of SCF and CXC-chemokine ligand 12 (CXCL-12) required for HSC maintenance in normal young and adult BM (1). Thus, HSCs reside in a hypoxic perivascular niche in which endothelial cells and stromal cells express distinct factors that promote HSC maintenance (1, 3).

## The niche of hematological malignancies

The contribution of the BM microenvironment in the leukemogenesis of leukemias and other hematopoietic neoplasms has been demonstrated in recent years (7). In hematological malignancies and with age, not only hematopoietic cells accumulate genetic alterations but also the HSC niche undergoes an extensive re-organization and thereby contributes to the generation of hematopoietic disorders such as leukemia (8). Nevertheless, despite these findings generated in experimental models, direct evidence that initial lesions in cells of the HSC niche trigger the development of the disease is still lacking for human leukemia.

In contrast, evidence that leukemic cells actively create an aberrant leukemic niche has been extensively generated in parallel to the description of the normal HSC niche. Normal HSCs and LSCs compete for the same region of the endosteal HSC niche (9–12). Through their localization in the normal HSC niche, LSCs co-opt evolutionary conserved signaling cascades of the BM microenvironment to further support the growth of the leukemia (7). In addition, malignant hematopoietic cells interact specifically with stromal and endothelial elements of the HSC niche to induce remodeling. As a consequence, the re-modelled BM microenvironment becomes supportive for AML LSCs and negatively affects healthy hematopoiesis and, specifically, HSCs (8–14). For example, genes in AML MSCs are in general hypomethylated which is accompanied by an altered expression of genes related to inflammation and CXCL-12 signaling (9). Similar results on the provision of pro-inflammatory signals by MSCs to promote leukemogenesis have been described in murine AML and CML models (10). Furthermore, pro-inflammatory cytokines such as interleukin (IL)-6 and TNF produced by leukemia cells have a central role in the promotion of disease development by promoting differentiation of leukemic progenitors and by simultaneous disruption and alteration of normal HSC function (8, 13, 14). Single cell RNA-sequencing further revealed that MSCs in AML have an impaired potential to differentiate into osteoblasts and to secrete key molecules required for HSCs maintenance such as CXCL-12 and SCF (9). The accumulation of these altered osteoprogenitors/osteoblasts, also characterized by increased production of inflammatory cytokines, modulates the endosteal HSC niche in a way that leukemogenesis is supported while the capacity to support normal hematopoiesis and HSCs is lost (11, 12).

Two recently published studies also highlighted the impact of alterations in the BM vasculature in the endosteal region for the maintenance of LSCs and the loss of HSCs. Increased permeability and reduced perfusion in the endosteal region resulted in hypoxic changes, the overproduction of ROS and nitric oxid, loss of barrier function and cell death. Inhibition of nitric oxid production reduced vascular permeability and preserved normal HSC function (15). Furthermore, enhanced inflammatory signaling in leukemia cells in the endosteal niche was associated with a reduction in vessels and HSCs (13). Importantly, a recent transcriptomic analysis revealed that pathologic crosstalk exists not only between AML and different niche cells but also within niche fractions (15).

The sympathetic nervous system (SNS) also promotes AML development. However, in contrast to normal hematopoiesis (16), nerves fibers get disrupted during AML development resulting in a reduction of HSC-promoting NG2^+^ niche cells and instruction of Nestin^+^ stromal cells to generate osteoblasts (17). Furthermore, recent preclinical work indicated a role for the pro-inflammatory cytokine IL-1β in the loss of sympathetic nerve fibers in the BM during MPN development. During early stages of the disease, JAK2V617F-expressing HSCs secrete IL-1β resulting in apoptosis of sympathetic fibers which was further associated with reduced numbers of Nestin-expressing MSCs and concomitant expansion of mutant HSCs (18). Overall, these studies indicate that LSCs, like HSCs, are dependent on signals derived from the HSC niche. Additionally, LSCs actively contribute to the re-organization of the niche from a microenvironment supportive for HSCs into an HSC unfriendly, aberrant leukemic microenvironment.

## Regulation of the HSC niche by immune cells in the BM

Self-renewal and maintenance of HSCs are not exclusively regulated by endothelial and stromal elements of the BM niche. As a primary and secondary lymphoid organ, the BM harbors a variety of different mature and immature cells of hematopoietic origin such as T cells, B cells monocytes/macrophages and neutrophils (7). Apart from developing and mature B cell subsets which occupy specific niches in the BM (19), most other lymphocyte subsets are widely distributed and can in general not be allocated to a specific location in the BM stroma or parenchyma (7). In addition, billions of leukocytes circulate every day through the BM. Recent studies highlighted the substantial contribution of multiple of these immune cell subsets in the regulation of the HSC niche (**Table 1**). Especially monocytes and macrophages but also different T cell subset have been shown to mediate the mobilization of HSCs indirectly by modulating MSCs (34–36, 39). Monocytes and macrophages comprise around 0.4% of total BM cells and are widely distributed throughout the BM.

**Table 1:** Immune cell subsets present in the BM stem cell niche and their reported effect on the HSCs and HSC niche function.

Cell Type	Frequency among total BM cells	Location in BM	Effect on HSC niche and/or HSCs	Effect on LSCs and leukemia niche
**Total CD4^+^ T cells**	~1%	Widely distributed throughout the BM	Antigen-activated CD4^+^ T cells modulate basal hematopoiesis (20). T helper cells actively regulate hematopoietic progenitor cell homeostasis (21). The interaction of HSPCs expressing immunogenic antigens with CD4^+^ T cells results in stem cell differentiation and depletion of immunogenic HSPCs (22).	-
**CD8^+^ T cells**	~0.5-1%	Widely distributed throughout the BM	-.	CD8^+^ CTLs are dysfunctional, fail to eliminate CML LSCs *in vivo* and rather promote their expansion (23, 24).
**Tregs**	~0.5%	Widely distributed but closely associated to the endosteum	Protect HSCs from immune destruction (25). Promote HSC maintenance through adenosine generation (26). Modulate lymphopoiesis *via* IL-7 derived ICAM-1^+^ stromal cells (27). Tregs regulate regulate myelopoiesis (28). Tregs regulate stromal cell function (29).	Protect LSCs from elimination by endogenous CD8^+^ CTLs and adoptively transferred CD8^+^ CTLs in CML (30) and AML (31), respectively. Treg depletion in a prophylactic setting (32), and in a minimal residual disease setting (33) promotes anti-leukemic immunity in a murine AML model.
**Neutrophils**	~35%	Widely distributed throughout the BM	Indirect *via* macrophages. Clearance of aged BM neutrophils by macrophages by promotes HSPC mobilization through reduction of CXCL-12 and CAR cells levels (34).	–
**Monocytes/** **macrophages**	~0.4%	Widely distributed throughout the BM	Regulate retention of HSCs and HSPCs indirectly by modulating MSCs and CXCL-12 expression in the BM (16, 35, 36). Promote the expansion of myeloid progenitors in response to microbial products (37).	Myeloid cells derived IL-6 and IL-1b modulate the niche in leukemia (18, 38). IL-6 induces myeloid differentiation of MPPs in CML (38). IL-1b induces apoptosis in sympathetic nerve fibers resulting in a loss of MSCs and expansion of HSCs in MPD (18).

## Regulatory T cells in hematopoiesis

The BM microenvironment does not only provide HSCs with key signals for survival, quiescence and self-renewal (1) but it also ensures protection against harmful signals that may drive HSCs differentiation, exhaustion or destruction (7). Tregs is one cellular subset, ideally suited to protect against the negative impact for example of inflammation by shaping an immune-suppressive environment. The BM is considered as a reservoir for Tregs in healthy individuals (40). Under homeostatic conditions, Tregs locate close to sinusoids and close to HSCs located at the endosteal surface in the BM (25) and comprise around 0.5% of all BM mononuclear cells and around one third of all CD4^+^ T cells in the BM (41). Thereby, Tregs are significantly enriched in frequency in the BM compared to other secondary lymphoid organs such as the spleen or the lymph nodes (40, 42). In contrast to blood and skin, BM Tregs are phenotypically characterized by the expression of CD45RA, C-X-C chemokine receptor type 4 (CXCR4) and the high expression of FOXP3 and CD25 (40, 43). Functionally, BM Tregs have a superior immunosuppressive activity compared to their blood counterparts (40). The BM retains these CXCR4-expressing Tregs through CXCL-12. After adoptive transfer into NSG mice, human Tregs preferentially homed into the BM which harbored elevated levels of CXCL-12. Blockade of the CXCR4/CXCL-12 interaction *via* monoclonal antibody (mAb) abolished the retention of Tregs in the BM but not the spleen (40). Therefore, it is speculated that the BM may represent a niche where naive Tregs accumulate to mature, survive, and/or undergo homeostatic proliferation while maintaining their naive phenotype. Furthermore, BM-resident Tregs may contribute to the formation of the immune-privileged perivascular HSC niche and shield the stem cell compartment from elimination by immune cells, excessive inflammation as well as cell-death.

Total CD4^+^ T cells (T helper cells and Tregs) secret hematopoietic-related cytokines and are essential for hematopoiesis stimulation during infection and hematologic recovery after BM transplantation [reviewed in (44)]. In addition, early observations that 1) STAT-4^–/–^ mice, which harbor predominately Th2 cells, have decreased numbers of cycling progenitors (21) and 2) that adoptive transfer of total CD4^+^ T cells into common gamma chain-deficient mice restored impaired myeloid differentiation indicated that CD4^+^ T cells regulate HSPC activity and basal hematopoiesis (20). These findings have been recently extended by Hernández-Malmierca and co-workers who demonstrated that the presentation of immunogenic antigens on MHC class II by HSPCs triggers an interaction with CD4^+^ T cells resulting in stem cell differentiation and depletion of immunogenic HSPCs (22).

**Figure 1:**
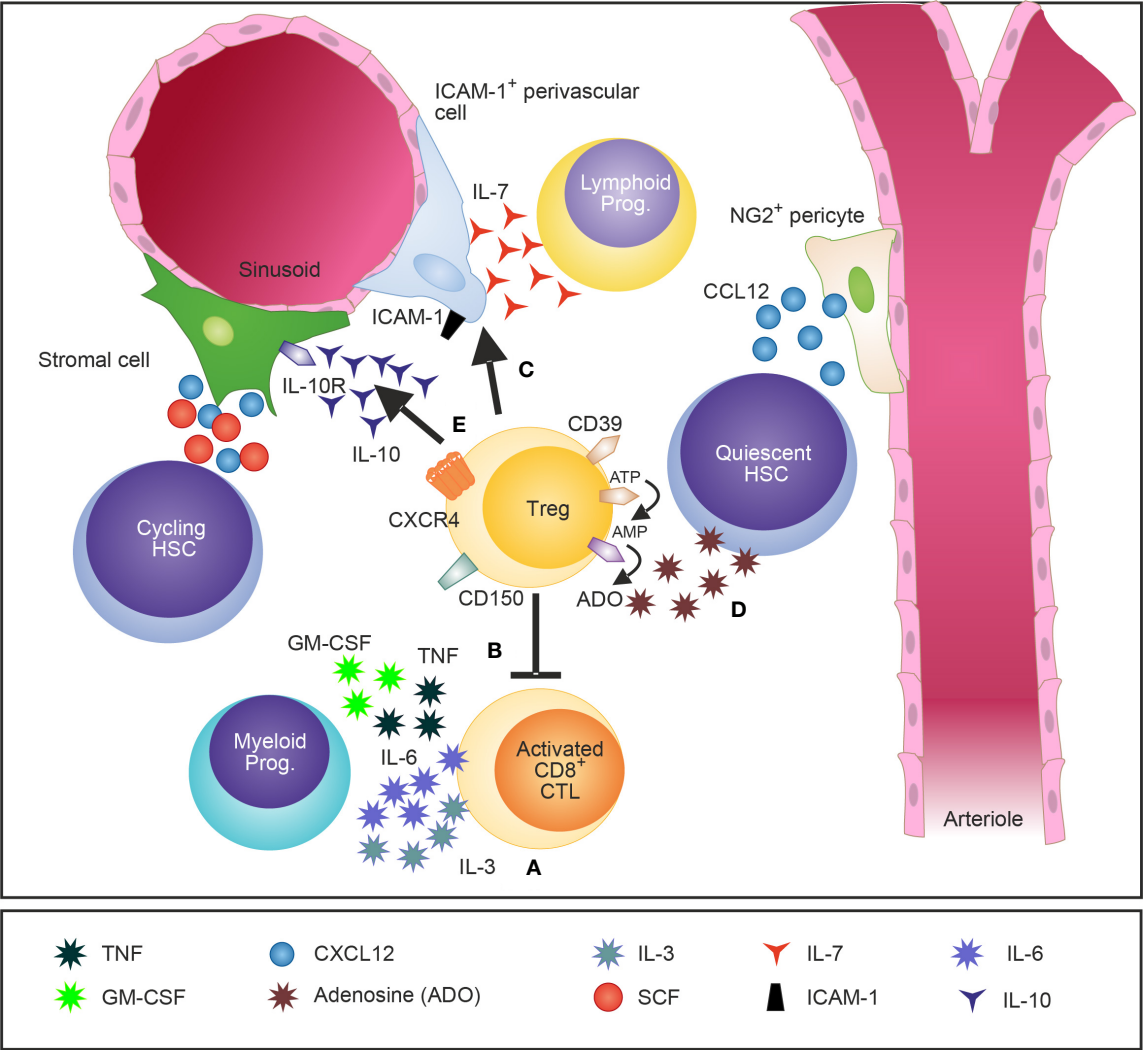
A schematic illustration of Tregs in the BM and their attributed effects on the HSCs niche and HSPCs. Tregs in the BM are widely distributed and localize close to the endosteum adjacent to HSCs. Tregs regulate IL-3 mediated differentiation of myeloid progenitors **(A)** (28) and modulate basal hematopoietic output by restricting production of myelopoiesis-promoting cytokine production by activated CD8 T cells in the BM **(B)** (45). Furthermore, Tregs control indirectly lymphopoiesis by mediating the release of the lymphoid cell growth and survival factor IL-7 by ICAM-1^+^ stromal cells of the HSC niche **(C)** (27). CD150^+^ Tregs were recently identified as crucial regulators of HSC quiescence and engraftment of HSCs into the BM after allogeneic BM transplantation **(D)** (26). In addition, IL-10 secreting Tregs have been shown to modulate the function of IL-10R-expressing BM stromal cells **(E)** (29).

Most of the effects observed upon transfer or depletion of total CD4^+^ T cells may most likely be mediated directly or indirectly by immunomodulatory Tregs. Tregs regulate IL-3 mediated differentiation of myeloid progenitors under homeostatic conditions. Depletion of Tregs *in situ* and co-transplantation of CD4^+^ FOXP3^+^ Tregs demonstrated that BM Tregs promote myeloid colony formation *in vitro* and myeloid differentiation of HSCs *in vivo (* 28). Importantly, the cognate interaction between Tregs and HSPCs was dependent on MHC class II expression suggesting a role for antigen-specific activation of Tregs in the regulation of HSCs in steady-state. In line with these initial findings, Kim et al. demonstrated that Foxp3 mutant scurfy mice have an enhanced granulopoiesis at the expense of B cell lymphopoiesis. However, this effect of myeloid differentiation was not mediated directly *via* Tregs but indirectly *via* the production of myeloid differentiation-related cytokines such as granulocyte-macrophage colony-stimulating factor (GM-CSF), TNF, and IL-6 by activated effector T cells (45). In addition, it was demonstrated that Tregs regulate the production of IL-7, a fundamental growth factor for lymphopoiesis, by intracellular adhesion molecule 1 (ICAM-1)-expressing perivascular stromal cells in the BM (27). In line with these findings, Camacho et al. recently demonstrated that BM Tregs regulate hematopoiesis indirectly through modulation of stromal cell function *via* secretion of IL-10 (29) (**Figure 1** and **Tables 1**, **2**). The role of Tregs in the regulation of stem cells, however, is not a unique feature for the BM and can be further extrapolated to multiple non-lymphoid tissues such as for example the skin, muscle, adipose tissue and brain (46–49). Consequently, accumulating scientific evidence supports the concept that Tregs control stem cells, their proliferation and self-renewal.

**Table 2:** Treg-mediated immunomodulatory mechanisms in the BM niche at steady state.

Reference	Target cell	Impact
Fujisaki (25)	Hematopoietic stem and progenitor cell	Tregs co-localize with HSPCs, generate an immune-privileged niche and support persistence of allogeneic HSCs.
Urbieta (28)	Myeloid progenitor cells	Tregs inhibit IL-3/SCF-mediated differentiation of myeloid progenitor through MHC-class II and TGF-β dependent mechanisms.
Kim (45)	T cell	Tregs indirectly regulate B cell lymphopoiesis by reducing the production the hematopoietic cytokines GM-CSF, TNF, and IL-6 by effector T cells.
Pierini (27)	Perivascular stromal cell	Tregs control normal B-cell lymphopoiesis by regulating the production of IL-7 by ICAM1^+^ perivascular stromal cells.
Camacho (29)	Mesenchymal stromal cell	BM Tregs regulate hematopoiesis indirectly through modulation of MSCs *via* secretion of IL-10.
Hirata (26)	Hematopoietic stem cell	Treg-derived adenosine protects HSCs from oxidative stress and maintains their quiescence. In addition, CD150^+^ Tregs, crucially contribute to the engraftment of HSCs into the BM after allogeneic BM transplantation.

In murine BM transplantation models, experimental data demonstrated that Tregs of the host not only suppresses graft-versus host disease but also favors hematopoietic reconstitution and HSC engraftment (50, 51). Similarly, co-transplantation of recipients alloantigen-specific Tregs together with HSC facilitated long-term hematopoietic reconstitution (52). In addition, Tregs activated *ex vivo* with allogeneic APCs also induced long-term tolerance and reconstitution of BM grafts in an antigen-specific manner *in vivo (* 53). A potential role for Tregs in hematopoiesis was further demonstrated in murine allogeneic BM transplantation experiments where depletion of host Tregs resulted in loss of engraftment of stem and progenitor cells to the BM suggesting that Tregs promote hematopoiesis and protect HSCs from elimination (25). In addition, a specific BM Treg subset characterized by the expression the surface markers CD150 and CD39 was recently identified as regulator of HSC quiescence and engraftment of HSCs into the BM after allogeneic BM transplantation. These BM Tregs reside near HSCs in the BM niche and protect HSCs from oxidative stress by secretion of adenosine. In addition, co-transfer of BM Tregs but not splenic Tregs together with allogeneic HSCs prevented the rejection of the graft (26). Mechanistically, long-term hematopoietic reconstitution has been shown to crucially depend on MHC-II signaling in a canine BM transplantation model as delivery of an anti-MHC-II mAb directly after BM transplantation prevented engraftment and hematopoietic reconstitution (54). In contrast, Huss and co-worker demonstrated in various murine allogeneic BM transplantation models that immunological but not hematopoietic recovery is impaired in recipient mice lacking MHC class II expression or treated with an anti-MHC II mAb (55) (**Figure 1** and **Table 1**).

In humans, the role of Tregs in HSC engraftment and long-term reconstitution after BM transplantation is still unclear. While depletion of total T cells from allogeneic BM resulted in rejection of grafts in a majority of the patients, the clinical benefit of Tregs in the graft still has to be demonstrated [reviewed in (50)]. Several clinical studies correlating the number of Tregs in the graft retrospectively with transplantation outcome delivered varying results and could not attribute a fundamental role of Tregs to long-term hematopoietic reconstitution. Similarly, conflicting data have been generated on the number of Tregs detected *in vivo* post-transplantation and transplantation outcome. Overall, these data imply a role for Tregs in the regulation of HSC function in mice while the role of Tregs on human hematopoiesis must still be validated.

## The adaptive immune system in leukemia

Experimental and clinical evidence indicates that myeloid leukemias are controlled by cells of the adaptive immune system to a certain extent (56, 57). Leukemic cells in myeloid malignancies are immunogenic, express HLA class I restricted leukemia epitopes and may be recognized by activated cytotoxic CD8^+^ T cells (CTLs) (7, 56, 57). LSCs and bulk leukemia cells express major histocompatibility and co-stimulatory molecules suggesting that LSCs can be recognized and interact directly with T cells (7, 56).

In addition, immune responses against myeloid leukemias as well as leukemia-antigen specific CD8^+^ CTLs have been observed in clinical and experimental studies (7, 56). However, activated CD8^+^ CTLs in myeloid leukemias are dysfunctional and fail to eliminate LSCs *in vivo (* 23, 24, 58–60). In CML, T cell exhaustion at diagnosis has been associated with an increased expression of immune inhibitory receptors and a reduced capacity to produce Th1-cytokines compared to patients in remission (59–63). Similar to CML, gene expression profiling of CD8^+^ CTLs from AML patients at diagnosis indicate that CTLs in intermediate and high risk AML are less functional than in favorable risk AML and exhibit an exhaustion immune signature (24). CD8^+^ CTL dysfunction was attributed to epigenetic silencing of activating immune checkpoint receptors rather than due to signaling by immune inhibitory immune checkpoint receptors (64). The phenomenon could in part explain the limited efficacy of antibodies that block inhibitory immune check-point inhibitors in AML. Furthermore, instead of promoting elimination, CD8^+^ CTL-related mechanism may also contribute to the expansion of LSCs and leukemia (23, 24). For example, secretion of the hematopoietic cytokine IL-3 has been shown to expand and maintain leukemia stem and progenitor cells in favorable risk AML patients, while AML cells in more aggressive forms of AML seem to develop largely independent of CD8^+^ T cell help (24). A systemic impairment/exhaustion of T cells and especially programmed cell death protein 1 (PD-1) and Eomes-expressing memory stem T cells has also been associated with relapse after allogeneic HSCT (65). Furthermore, response to chemotherapy correlated with the immune signatures indicative for the restoration of T cell function (58).

In contrast to CD8^+^ T cells, the role of CD4^+^ T cells in the control of leukemia has been studied less intensively (7). Nevertheless, even though less studied, several independent reports demonstrated that myeloid leukemia cells are competent to process and present endogenous immunogenic leukemia peptides in the context of HLA class II resulting in leukemia antigen-specific CD4^+^ T cell response [reviewed in (7)]. Recently, the HLA class I and class II ligandome was characterized using immunoprecipitation and mass spectrometry in CD34^+^ stem/progenitor cells from AML and CML patients and CD34^+^CD38^-^ healthy controls (66, 67). For AML, the analysis revealed 36 AML-specific leukemia antigens. Importantly, the top-ranked HLA class II leukemia antigens were successfully validated in T cell activation assays *in vitro (* 67). A similar analysis for CML identified 44 CML-associated HLA class II antigens (66). A clinical vaccination study which assessed the immunogenicity and anti-tumor activity of a vaccine cocktail comprising 4 b3a2-petides restricted to HLA I and one b3a2-petide restricted to HLA II in b3a2-espressing CML patients with stable measurable residual disease, demonstrated immunological activity of the vaccine as illustrated by CML-peptide specific CD4^+^ T cells *in vitro* in 13 of 14 evaluable patients (68). Furthermore, vaccination of a 63-year old woman which displayed residual CML cells after cessation of IFN-α therapy with a immunogenic b2a2 breakpoint derived peptide able to bind several HLA-DR molecules induced a complete molecular response in blood and BM (69). Overall, these data suggest that leukemia-antigen specific CD4^+^ T cells may potentially also contribute to anti-tumoral immunity in leukemia.

## Immune escape of leukemia and LSCs

In AML, T cells appear to infiltrate the BM microenvironment, but their efficacy to control leukemia and eliminate LSCs is limited. In general, the BM microenvironment in AML may be considered as immune cold (70, 71). Analysis of an 18-gene tumor inflammation signature in solid tumor and AML samples using data from the Cancer Genome Atlas revealed that AML patients have a low expression of this signature which is associated with an immune cold microenvironment (70). Similarly, transcriptomic, and proteomic analysis of AML BM cells demonstrated that 70% of AML patients display an immune-depleted signature (71). Because LSCs reside primarily in the BM, the generation of an immunosuppressive/immune cold environment may be considered as a central mechanism how LSCs escape immune control. However, several different mechanisms may contribute to generation of an immune cold environment and the subsequent immune escape in myeloid leukemia.

### Immune checkpoint ligands/receptors

One mechanism how LSCs can escape immunosurveillance is through the activation of immune checkpoint pathways. Immune checkpoint pathways play a fundamental role in the regulation of immune homeostasis (72). The balance between stimulatory and inhibitory signals determines the amplitude and quality of the antigen-specific T cell responses (72). Immune checkpoint inhibitors targeting the co-inhibitory molecules cytotoxic T-lymphocyte-associated protein 4 (CTLA-4) and PD-1 have been approved for the treatment of various solid tumors and lymphoma (72). Increased expression of the inhibitory ligands for T-cell–regulating checkpoints such as CTLA-4, PD-1, B7-H3 and T cell immunoglobulin and mucin-domain containing-3 (TIM-3) was reported in AML and was associated with T cell exhaustion and poor prognosis (62, 63, 73). This CD8^+^ T cell exhaustion was in part reversible by blocking the immune checkpoint molecules PD-1, CTLA-4, and TIM-3 (63) or by OX-40 co-stimulation (62). However, in patients, immune checkpoint inhibitors against CTLA-4 and PD-1 seem to be less effective in AML compared to solid tumors (74). One possible explanation for this lack of potency in AML is that BM-infiltrating T cells in AML expressing various inhibitory receptors, such as TIM-3 and lymphocyte-activation gene 3 (LAG-3). Another explanation could be the limited or restricted surface expression of PD-L1 in *de novo* AML (73, 75) and variable kinetic of PD-L1 expression during the progression of the disease. For example, PD-L1 expression increases on AML cells during relapse and could represent an adaptive immune escape mechanism (74). However, it also unclear whether the increased expression of immune inhibitory receptors reflects T cell exhaustion or whether it is indicative for differentiated effector T population. Currently several clinical trials with different antibodies targeting the PD-1/PD-L1 interaction are ongoing in combination with CTLA-4 inhibition, in combination with chemotherapy, or in combination with hypomethylating agents that upregulate the expression of PD-L1 on leukemia cells (76).

### Tregs in myeloid leukemia

Because the efficacy of immune checkpoint inhibitor treatments in myeloid malignancies is limited, other immune-regulatory mechanisms in the BM may be in place. Studies in mice and humans identified the accumulation of Tregs as a potential major immune-regulatory mechanism in myeloid leukemia. Several clinical studies reported increased frequencies of Tregs in blood and/or BM at diagnosis, after allogeneic hematopoietic stem cell transplantation (HSCT) or during induction chemotherapy in myeloid leukemias (77, 78).

#### Tregs in CML

In CML, numbers and frequencies of effector Tregs in peripheral blood and BM are also increased in CML patients at diagnosis and in patients refractory to tyrosine kinase inhibitor (TKI) treatment (59–61). These effector Tregs are characterized by the expression of the immune checkpoints PD-1, TIM-3 and CTLA-4, all markers for an increased suppressive activity (61). Anatomically, Tregs in CML are widely distributed throughout the BM parenchyma in mice and humans. In human CML, the majority of Tregs were localized close to CD8^+^ CTLs and not close to CD34^+^ CML stem/progenitor cells (30). Treatment with the TKIs Imatinib, Dasatinib and Nilotinib demonstrated an inhibitory effect of these TKIs on the quantity of Tregs in CML patients. Tregs were especially reduced in patients who achieved a complete cytogenetic response (59, 79). Similarly, Imatinib-treated CML patients in complete molecular remission (CMR) displayed a selective depletion of effector Tregs which was accompanied by an increase in effector/memory CD8^+^ CTLs in contrast to CMR patients who did not reach a complete molecular remission (80). Furthermore, a successful maintenance of treatment-free-remission (TFR) is associated with reduced numbers of Tregs (81–83). In the frame of the JALSG-STIM213 trial, the frequency of effector Tregs in the peripheral blood has been identified as potential biomarker for TFR after Imatinib stop (84). Importantly, tyrosine kinase inhibitor (TKI) treatment responses were more potent in patients with less exhausted T cells (60). Because the state of T cell dysfunction at diagnosis is reverted in patients that reach a deep molecular response (60), one may hypothesize that off-target immune-modulatory effects of TKIs trigger already pre-existing anti-tumoral immune responses or interfere with immunosuppressive cells as Tregs. In solid tumors, tyrosine kinase inhibitor (TKI) treatment with Imatinib and Dasatinib has been shown to reduce immunosuppressive immunoindolamine-2, 3-dioxygenase (IDO) expression, a key factor in the generation of Tregs, and to increase the numbers of intratumoral CD8^+^ CTLs (85, 86). Future therapeutic approaches may therefore aim at an expansion and effector function of CTLs through targeting of Tregs. For example, Tregs expressing the tumor necrosis factor 4 (TNFRSF4, alias OX-40) have recently been identified as the key regulator of immune escape of CML LSCs in mice (30). Stimulation of Tnfrsf4-signaling did not deplete Tregs but reduced the capacity of Tregs to protect LSCs from granzyme-mediated CD8^+^ CTL- killing in a murine CML model.

#### Tregs in AML

**Figure 2:**
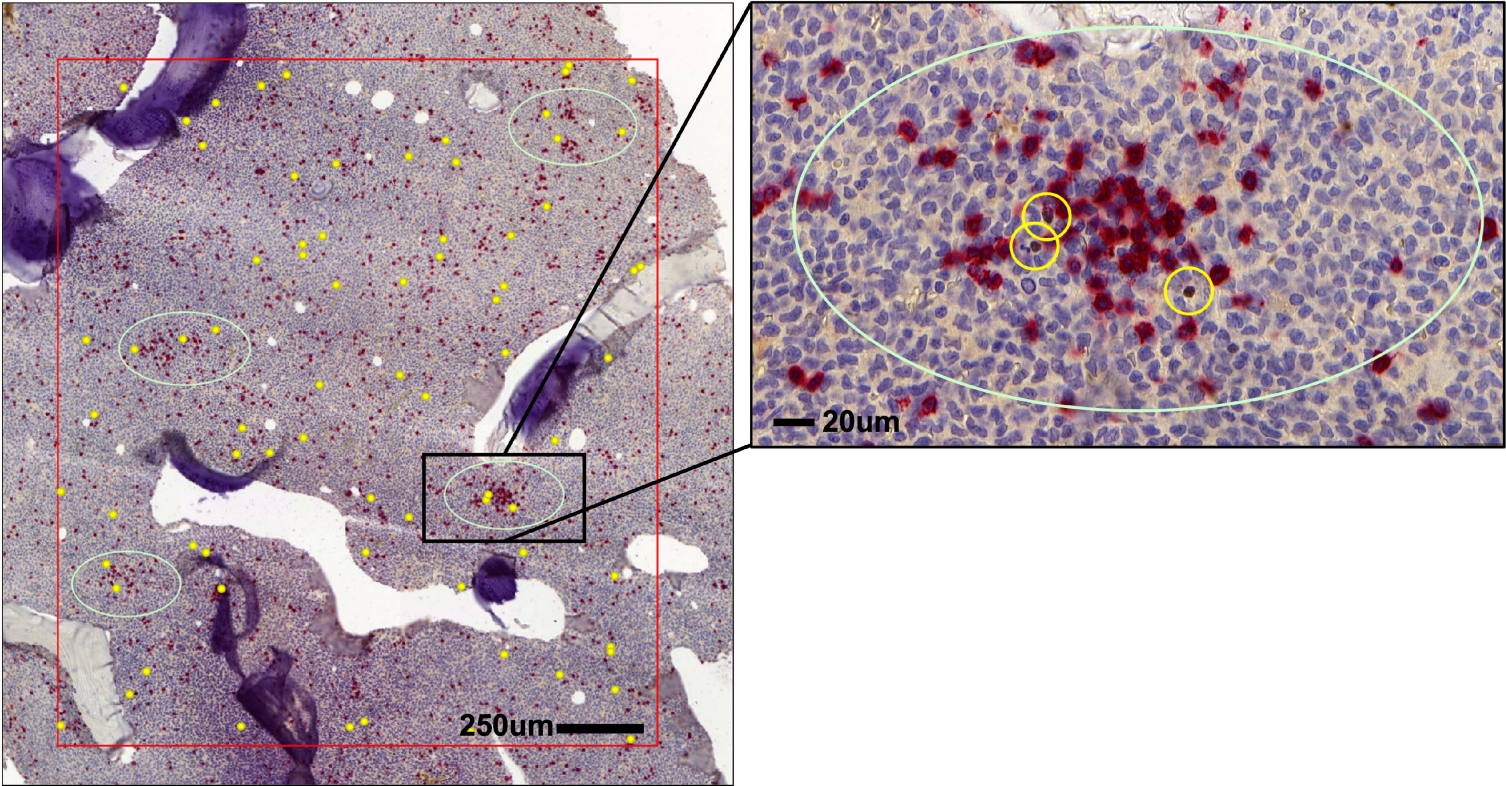
Distribution of Tregs and CD8^+^ T cells in AML bone marrow (BM). Spatial localization of FOXP3^+^ Tregs (brown, yellow circles) in respect to CD8^+^ T cells (red) in the BM of AML patients.

Like in CML (30), Tregs in the BM of AML patients are widely distributed throughout the BM parenchyma and are occasionally located close to clusters of CD8^+^ T cells (**Figure 2**). Tregs frequencies in blood and BM are significantly elevated in adult and pediatric AML patients compared to Tregs from healthy donors and further increase at relapse (87–89). In general, increased levels of Tregs at diagnosis correlate with poor response to induction chemotherapy and relapse after allogeneic HSCT (78, 90). In contrast to baseline AML biopsies, higher numbers of Tregs early after induction therapy are associated with higher complete remission rates and better overall survival. This discrepancy may be attributed to the different cellular composition of the BM microenvironment early after chemotherapy and at an advanced stage of the disease. Furthermore, a phase IV clinical trial (NCT01347996) which assessed the efficacy of immunotherapy with histamine dihydrochloride (HDC) and low-dose IL-2 in the post-consolidation phase in AML 84 patients in first complete remission demonstrated an association of AML relapse with HDC/IL-2 treatment-induced accumulation of natural Tregs in the blood (91). Therefore, Tregs could either serve as immune-modulators and support hematopoietic recovery or induce immune escape of leukemia by limiting CTL activity. On the other hand, Tregs seem to localize close to HSCs in the niche during homeostasis and may therefore directly facilitate their homing in the BM and emergency hematopoiesis.

In contrast to CD8^+^ T cells, Tregs from AML patients are not exhausted but rather hyper-functional as illustrated by a higher migratory and immunosuppressive potential than Tregs from blood and BM of healthy volunteers (37). However, even though, the prevalence of Tregs in the BM and blood in AML is identical (92), BM Tregs are more suppressive than Tregs derived from blood of AML patients (77). Phenotypically, BM Tregs of AML patients express the memory marker CD45RO along with CTLA-4 and CD95 and secret low levels of the immunosuppressive cytokines IL-10 and TNF. Furthermore, BM Tregs in AML have a high turnover compared to BM Tregs from healthy donors as indicated by 8-fold higher proliferation and 4-fold higher apoptosis rate (93).

**Figure 3:**
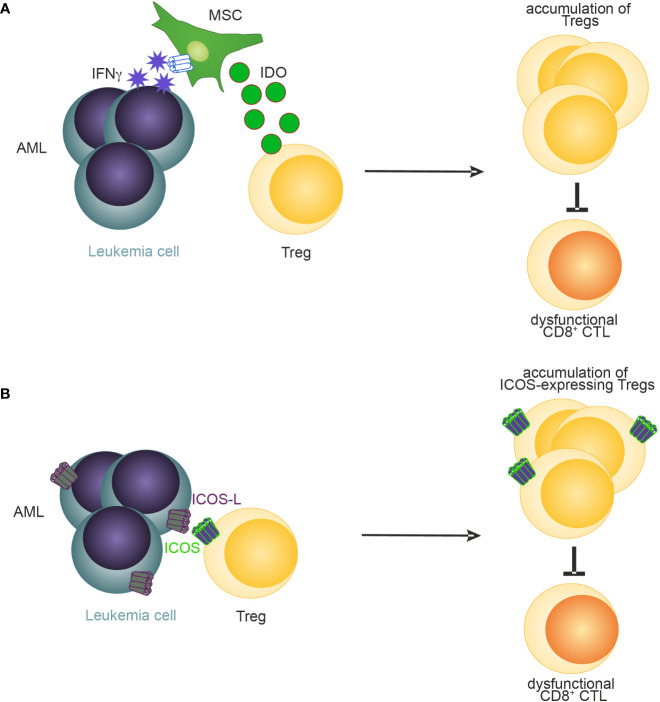
A schematic illustration of reported mechanisms leading to accumulation of Tregs in AML. **(A)** MSC-derived IDO (94) and **(B)** ICOS-ICOSL signaling (97) promotes the accumulation of Tregs in AML.

Recently, several mechanisms have been identified that promote the expansion and BM retention of Tregs in AML (37, 94–96). For example, Tregs in blood and BM of patients have been shown to express elevated levels of CXCR4 on the cell surface compared to Tregs derived from healthy donors suggesting that, like in homeostasis, the BM retains CXCR4-expressing Tregs through CXCL-12 in AML (37). Similarly, the frequency of Tregs expressing inducible T-cell co-stimulator (ICOS) has been negatively associated with outcome in AML patients (97). Co-simulation of ICOS signaling through the provision of ICOS-ligand by AML cells expanded Tregs and maintained their suppressive function in a murine C1498 AML model (97) (**Figure 3**). Another mechanism how Tregs may be expanded and sustained in AML is through IFN-y induced secretion of IDO by mesenchymal stromal cells (94) (MSCs, **Figure 3**). Blockade of IFN-y production by AML cells lowered IDO1 expression resulting in reduced Treg infiltration and leukemia engraftment. Furthermore, daunorubicin-treated AML cells induce IDO1 secretion in dendritic cells (DCs) resulting in the accumulation of PD-1-expressing Tregs (98). In the BM of newly diagnosed AML patients, high IDO expression in MSCs was associated with elevated levels of Tregs. Furthermore, an IDO1-related immune gene signature has been identified as a negative predictor for overall survival in AML (99). Similarly, MSCs derived from Fanconi anaemia patients with AML secrete high levels of prostaglandins which resulted in Treg induction and subsequent inhibition of CD8^+^ CTLs (100). Lastly, PD-L1 expression on AML cells have been recently demonstrated to promote the conversion and expansion of PD-1^+^ Tregs from conventional CD4^+^ T cells (95).

However, the experimental and clinical evidence that directly links Tregs with T-cell anergy/exhaustion in AML is still limited and mostly restricted to pre-clinical mouse models. For example, Tregs have been shown to inhibit the function of adoptively transferred AML-specific CTLs. Depletion of Tregs during adoptive transfer of CTLs improved survival of AML mice (31). Similarly, Treg depletion prior to leukemia induction has been shown to trigger an endogenous anti-leukemic CTL response resulting in prolonged survival of MLL-AF9 AML mice (32). Unpublished data from our group and a recent publication by Hasegewa and co-workers support the notion that Tregs in MLL-AF9 mice limit anti-leukemic immunity only in a situation with minimal leukemia load but not in an advanced stage of the disease (33). Similar to human AML (37), BM Tregs in MLL-AF9 mice have shown to express elevated levels of CXCR4 on the cell surface. Blockade of the CCL3/CCR1-CCR5 and CXCL-12/CXCR4 axis prevented the accumulation of Tregs in BM of MLL-AF9 AML mice (32). These data indicate that Tregs create an immunosuppressive microenvironment in the BM thereby reducing CTL function and immunosurveillance of AML cells. In addition, *in vitro* experiments revealed that AML cells themselves secret soluble factors that restrain T cell and NK cell function (101). Of note, Hasegawa et al. using the same AML model and RAG-knockout mice demonstrated that CTLs indeed have the capacity to eradicate leukemia at an early stage of the disease (low leukemia load), whereas antigen-specific CTLs are exhausted in animals with advanced leukemia (33).

However, Tregs in AML may not only hamper anti-leukemic T cell immunity but may also directly affect the survival and function of AML blasts LSCs. It was recently demonstrated that Treg-derived IL-10 promotes stemness of murine and human AML LSCs through activation of PI3K/AKT signaling pathway (96). Similarly, the cytokine IL-35 produced by Tregs promotes the proliferation of AML blasts (102).

Even though leukemia-antigen specificity of CD4^+^ T cells have been documented in several independent studies (7, 56), whether AML antigen–specific Tregs are part of this CD4^+^ T cell population is still unclear. In general, the evidence for functional tumor antigen–specific Tregs in cancer is very weak due to the lack of adequate MHC class II tetramers, and antigen-specific Tregs have only been documented in a few solid tumors and in B acute lymphoblastic leukemia (51, 103). Nevertheless, recent findings of a pre-clinical study suggests that antigen stimulation may play an important role in the activation and accumulation of Tregs in CML BM (30).

Overall, these studies imply that LSCs reside in an immune-privileged niche in the BM which harbors multiple immune-regulatory mechanisms. It may be assumed that the accumulation of Tregs in the BM represents one immune-regulatory mechanism in leukemia which limits immunosurveillance of LSCs and thereby contributes to disease progression and LSC maintenance. However, why Tregs accumulate in the BM, how this can be controlled, whether they are leukemia antigen-specific and whether selective targeting of Tregs in myeloid neoplasm (e.g. *via* an agonistic TNFRSF4 antibody) can induce immune control in humans is still unclear.

### Altering the expression of major histocompatibility complex (MHC) molecules

The elimination of cancer/leukemia cells depends on tumor antigen expression on MHC class I and II. In AML, mutations and immune escape mechanisms may result in the downregulation of MHC expression on leukemia cells. However, reduced, or lost MHC expression has been primarily observed on AML cells after allogeneic HSCT. For example, in 17 out of 34 patients, AML cells displayed a reduced expression of MHC class II at relapse after allogeneic HSCT compared to diagnosis. The downregulation of MHC was triggered by epigenetic mechanisms and could be restored by administration of IFN-y (104). Similarly, another study profiling immune signatures in AML cells after allogeneic HSCT identified the downregulation of MHC class II on a transcriptional level as a major immune escape mechanism (105). Furthermore, impaired processing and loading of leukemia-associated antigen on MHC class II has been described as potential immuno-editing process in AML.

## Therapeutic strategies targeting Tregs

Targeting signaling cascades that promote Treg-mediated immunosuppression in myeloid leukemia or Treg-specific surface antigens may be a viable approach to block Treg function or deplete Tregs and induce anti-leukemic immunity.

### CTLA-4

The anti-CTLA-4 mAb ipilimumab has been approved for the treatment of metastatic melanoma. However, despite the increased expression of CTLA-4 on Tregs in the tumor microenvironment (TME) of cancer patients and pre-clinical evidence showing the capacity of ipilimumab to deplete Tregs *via* ADCC-dependent mechanisms, ipilimumab is thought to induce infiltration of intratumoral CD4^+^ and CD8^+^ effector T cells without depleting Tregs in the TME in humans (106). In addition, the specificity of CTLA-4 as a Treg restricted target is widely debated because CTLA-4 is widely expressed on activated CD4^+^ T helper and CD8^+^ effector T cells and monocyte-derived DCs (72). To ensure a controlled activity of the antibody in the TME and to decouple antitumor efficacy from immunotherapy-related toxicity, a CTLA-4 dual variable domain immunoglobulin (anti-CTLA-4 DVD) was developed where the inner anti-CTLA-4 binding domain is shielded by an outer tumor-targeting domain (106). In the TME, the presence of activated membrane type-serine protease 1 results in cleavage of the outer domain and the liberation of the CTLA-4 binding site. To enhance target specificity, currently bi-specific antibodies targeting CTLA-4 together with other surface receptors with increased expression on Tregs are designed and evaluated. OX-40 is highly expressed on Tregs in AML (60) and CML (30) and is currently studied as a treatment for patients with refractory/relapsed AML as monotherapy or in combination with azacitidine and avelumab (NCT03390296). A human CTLA-4 x OX-40 bispecific antibody (ATOR-1015) which demonstrated efficacy in Treg depletion assays *in vitro* and syngeneic mouse models *in vivo (* 106) is currently under evaluation in a first-in-human phase I clinical trial (NCT03782467). Similarly, a humanized CTLA-4 x GITR bispecific antibody (ATOR-1144) was shown to deplete Tregs *in vitro* through ADCC-dependent mechanisms (107).

Clinical trials assessing the potential of targeting CTLA-4 in AML are limited. In a phase 1b study, ipilimumab was tested in 522 patients with hematologic malignancies relapsing after allogeneic HSCT. The complete response rate was 23%. In addition, immune-related adverse events and graft-versus-host disease were reported (108). Ipilimumab is currently evaluated in several clinical phase I trials as a treatment for relapsed/refractory AML patients (NCT03912064, NCT03600155, NCT01822509) and as maintenance therapy for AML and MDS patients alone or in combination the anti-PD-1 antibody Nivolumab and/or azacitidine after allogeneic HSCT (NCT02846376) and for its potential to induce graft-versus-malignancy effects after allogeneic HSCT (NCT00060372).

### CD25

CD25 is highly expressed on activated Tregs in the TME and only marginally on effector T cells (109). Therefore, several therapeutics targeting CD25 have been recently developed and were approved for the prevention of organ transplantation rejection (anti-CD25 mAb Basiliximab) and the treatment of cutaneous T-cell lymphoma (IL-2-diphtheria toxin fusion protein denileukin diftitox). However, clinical studies have demonstrated that denileukin diftitox is ineffective in eliminating Tregs in patients (107). An open-label, multicenter Phase I clinical trial is underway investigating the safety and tolerability of the anti-CD25 depleting antibody RO7296682 in patients with advanced solid tumors (NCT04158583). In addition, the potential of basiliximab conjugated with 1,4,7,10-tetraazacyclododecane tetraacetic acid (DOTA) and radiolabeled with Yttrium-90 given together with fludarabine, melphalan, and total marrow and lymphoid irradiation (TMLI) is evaluated for the treatment of patients with high-risk acute leukemia or myelodysplastic syndrome (NCT05139004). A phase I dose-escalation clinical trial assessed safety and efficacy of Camidanlumab tesirine, an antibody-drug conjugate targeting CD25 in 34 relapsed/refractory AML patients (110). Two patients achieved complete responses with incomplete hematologic recovery. However, the trial was terminated early for reasons other than safety and efficacy.

### FOXP3

FOXP3 serves as a lineage-specific transcription factor of Tregs. As FOXP3 is primarily expressed in the nucleus, FOXP3 has never been considered as a viable target to deplete Tregs in cancer. However, the potential of targeting FOXP3 directly has recently gained considerable interest. A T cell receptor mimic antibody recognizing a FOXP3-derived epitope in the context of HLA-A*02:01 was shown to selectively recognize and eliminate FOXP3-expressing Tregs in a PBMC xenograft model (111). Similar results were obtained when the TCR mimic antibody was engineered in a bispecific T-cell engager. Another strategy currently followed to selectively deplete Tregs in cancer are antisense oligonucleotide against FOXP3. The clinical candidate antisense oligonucleotide AZD8701 demonstrated encouraging efficacy in reducing FOXP3 and Treg immunosuppressive function in primary human Tregs *in vitro* as well as in humanized mouse models *in vivo (* 112). The safety and tolerability of AZD8701 is currently evaluated in a phase 1a/b clinical trial with or without Durvalumab in patients with advanced solid tumors (NCT04504669). However, strategies targeting FOXP3 bear the risk for the development of autoimmune disease as FOXP3 expression is most likely not limited to tumor tissues. Clinical trials targeting FOXP3 in myeloid malignancies are pending.

## Concluding remarks

The BM is a hematopoietic organ that harbors in addition to classical niche cells also a variety of different immune cells such as B and T cells. CD4^+^ T cells including Tregs significantly shape the cytokine milieu in the BM microenvironment and thereby promote key functions of HSC such as quiescence, self-renewal proliferation and differentiation. These mechanisms evolved to guarantee a constant generation of blood cells from all lineages and to prevent damage and exhaustion of the stem cell pool. LSCs co-opt these evolutionary conserved mechanisms of the HSC niche and at the same time re-model the HSC niche to promote leukemogenesis and LSC function and to reduce the HSC reservoir in the BM. In leukemia, Tregs regulate the expansion of LSCs and leukemia progenitors and contribute to immune evasion of leukemia cells by cell-interactions or through secretion of cytokines. Therefore, targeting Tregs seems to be an attractive therapeutic option and warrants further investigation in humans. So far, however, hardly any clinical information on the potential of targeting Tregs in myeloid leukemia is available. Only a limited number of clinical trials currently addresses this possibility mainly by targeting immune-related surface receptors such as CD25, CTLA-4 and TNFRSF4 which are expressed on Tregs. However, whether these approaches will lead to the elimination of LSCs and thereby cure of leukemia is still unknown. Similarly, our knowledge on the emergence of severe adverse events such as the induction of inflammation or auto-immune disease upon Treg manipulation and their effect on the disease is limited and must be crucially considered prior to initiating and planning clinical trials. Consequently, a better understanding of immune cells including Tregs and their function in the BM microenvironment during disease and homeostasis may contribute to the generation of novel and safe immunotherapeutic strategies aiming at elimination of leukemia at the level of the LSC and the identification of factors and interactions which may translate into the possibility to generate cells of different hematopoietic lineages ex vivo for specific applications, respectively.

## Author contributions

CR conceptualized and wrote the review article.

## Acknowledgments

This work was supported by grants from the Swiss National Science Foundation (310030_179394) and the Stiftung für klinisch-experimentelle Tumorforschung. I thank Adrian F. Ochsenbein for helpful discussions and input during the preparation of the manuscript and Stefan Forster for generating the IHC images. I apologize to the authors whose work was not mentioned due to space limitations.

## Conflict of interest

The author declares that the research was conducted in the absence of any commercial or financial relationships that could be construed as a potential conflict of interest.

## Publisher’s note

All claims expressed in this article are solely those of the authors and do not necessarily represent those of their affiliated organizations, or those of the publisher, the editors and the reviewers. Any product that may be evaluated in this article, or claim that may be made by its manufacturer, is not guaranteed or endorsed by the publisher.
